# Clinical significance of R‐wave amplitude in lead V_1_ and inferobasal myocardial infarction in patients with inferior wall myocardial infarction

**DOI:** 10.1111/anec.13114

**Published:** 2024-04-02

**Authors:** Xiao‐Bin Zheng, Hai‐Yan Wu, Ming Zhang, Bing‐Qi Yao

**Affiliations:** ^1^ Department of Cardiology Shanxi Cardiovascular Hospital Taiyuan China

**Keywords:** inferior wall myocardial infarction, QRS duration, risk stratification, R‐wave amplitude in V_1_

## Abstract

**Objective:**

To assess electrocardiogram (ECG) for risk stratification in inferior ST‐elevation myocardial infarction (STEMI) patients within 24 h.

**Methods:**

Three hundred thirty‐four patients were divided into four ECG‐based groups: Group A: R V_1_ <0.3 mV with ST‐segment elevation (ST↑) V_7_–V_9_, Group B: R V_1_ <0.3 mV without ST↑ V_7_–V_9_, Group C: R V_1_ ≥0.3 mV with ST↑ V_7_–V_9_, and Group D: R V_1_ ≥0.3 mV without ST↑ V_7_–V_9_.

**Results:**

Group A demonstrated the longest QRS duration, followed by Groups B, C, and D. ECG signs for right ventricle (RV) infarction were more common in Groups A and B (*p* < .01). ST elevation in V_6_, indicative of left ventricle (LV) lateral injury, was more higher in Group C than in Group A, while the ∑ST↑ V_3_R + V_4_R + V_5_R, representing RV infarction, showed the opposite trend (*p* < .05). The estimated LV infarct size from ECG was similar between Groups A and C, yet Group A had higher creatine kinase MB isoform (CK‐MB; *p* < .05). Cardiac troponin I (cTNI) was higher in Groups A and C than in B and D (*p* < .05 and *p* = .16, respectively). NT‐proBNP decreased across groups (*p* = .20), with the highest left ventricular ejection fraction (LVEF) observed in Group D (*p* < .05). Group A notably demonstrated more cardiac dysfunction within 4 h post‐onset.

**Conclusions:**

For inferior STEMI patients, concurrent R V_1_ <0.3 mV with ST↑ V_7_–V_9_ suggests prolonged ventricular activation and notable myocardial damage. RV infarction's dominance over LV lateral injury might explain these observations.

## Graphical abstract

R‐wave amplitude <0.3 mV in lead V_1_ concomitant with ST‐segment elevation in leads V_7_–V_9_ predicts a larger infarct size and longer ventricular activation time in patients with inferior myocardial infarction.

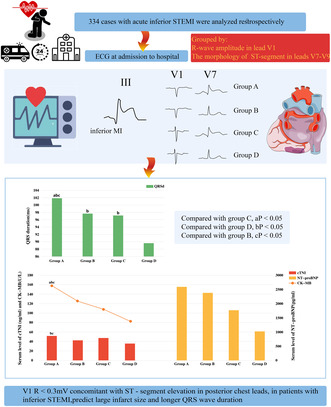

## 1 INTRODUCTION

Acute myocardial infarction (AMI) is a critical condition in coronary heart disease. An electrocardiogram (ECG) plays a pivotal role in its early identification and differential diagnosis. Among AMI cases, ST‐elevation myocardial infarction (STEMI) constitutes 25%–40%, and about 58.3% of STEMI instances are inferior STEMI (Gupta et al., 2020; O'Gara et al., 2013). In the context of STEMI, a pathological Q wave on the ECG prior to reperfusion is commonly noted. The prevalence of Q wave during the thrombolytic therapy era was between 33% and 53%, and during the percutaneous coronary intervention (PCI) era, it was between 21% and 72% (Andrews et al., 2000; Armstrong et al., 2009; Raitt et al., 1995; Topal et al., 2017, 2020). Notably, in STEMI, an abnormal Q wave might not conclusively indicate irreversible myocardial damage. It could signify intense, yet reversible, cellular ischemia or depict stunned and hibernating myocardium (Bateman et al., 1983; Sztajzel & Urban, 2000). Even though a baseline Q wave suggests a poor prognosis, STEMI patients with a Q wave still gain advantages from primary PCI (Topal et al., 2017), and effective reperfusion therapy can lead to Q‐wave regression or the return of R wave from hours to weeks post‐STEMI, hinting at recovered myocardial and improved left ventricular functions (Rijnierse et al., 2012; Sztajzel & Urban, 2000).

In the chronic phase of inferior STEMI, a pronounced R wave in lead V_1_ is indicative of left ventricle (LV) lateral wall myocardial necrosis, often seen as a “pathological R wave” mirror reflecting the lateral wall Q wave (Bayés de Luna et al., 2015). It is worth noting that even without an infarction, significant myocardial ischemia can produce early QRS changes. Our clinical observations confirm that some patients with an inferior myocardial infarction (IMI) present an obvious R wave in lead V_1_ shortly after onset, and moreover, an accentuated R wave during the acute phase may evolve into a more conspicuous R wave as the condition becomes chronic. This observation has led us to speculate that a significant R wave in lead V_1_ could be indicative of substantial lateral wall myocardial injury in cases of IMI. Additionally, right ventricular myocardial infarction (RVMI) can induce QRS alterations in the precordial leads (Halkett et al., 1986; Roesler & Dressler, 1947). Given the anatomical positioning of the right ventricle (RV) against the lateral wall of LV and based on ECG vector cancellation theory, RVMI can potentially offset the ischemic vector from the lateral wall. Thus, the presence of RVMI might suppress the R‐wave amplitude in lead V_1_, possibly masking the lateral myocardial damage.

ST‐segment elevation (ST↑) in leads V_7_–V_9_ suggests a concurrent inferobasal myocardial infarction (IBMI), implying a greater myocardial injury (Adawi & Atar, 2008). And a prolonged QRS duration (QRSd), caused by widespread ischemia from multivessel disease, is also a predictor of worse outcomes in AMI patients (Shah et al., 2016). Consequently, this study seeks to explore the implications of R‐wave amplitude in lead V_1_, ST‐segment patterns in posterior chest leads, and their impact on QRSd. The goal is to refine our understanding of the ECG's utility in stratifying risks for patients diagnosed with inferior STEMI.

## 2 METHODS

### 2.1 Study population

**FIGURE 1:**
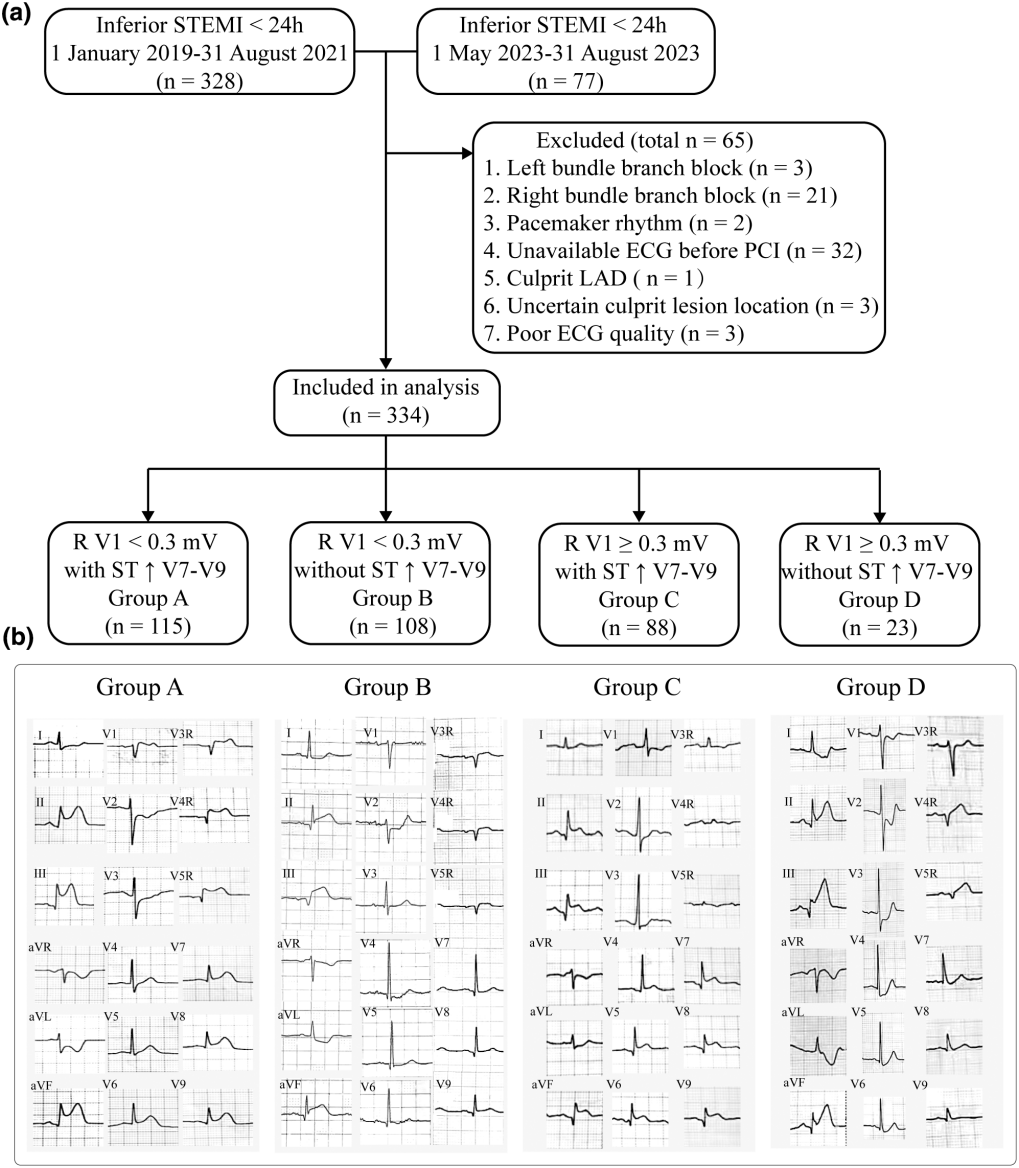
Flow chart of patient inclusion (a) and the ECG examples in four groups (b).

This retrospective study at Shanxi Cardiovascular Hospital involved 334 patients with acute inferior STEMI, defined in accordance with the Fourth Universal Definition of Myocardial Infarction, including elevated cardiac troponin I (cTNI) levels, symptoms of myocardial ischemia, and new ST↑ in the inferior ECG leads. (Thygesen et al., 2018). The patients undergoing emergency coronary angiography were included from January 2019 to August 2021 and May 2023 to August 2023 to maintain sample size (Figure 1a). Reperfusion within 12 h is more beneficial, but guidelines suggest extending the window to 24 h is also viable, so our criteria include up to 24 h (Lawton et al., 2022). Clinical data and in‐hospital outcomes were extracted from electronic medical records, selecting the peak levels of cardiac enzymes and troponin during their stay. Exclusions included patients with: (1) concurrent acute anterior STEMI or those with the left anterior descending (LAD) artery as the culprit vessel, (2) other heart conditions such as myocarditis, pericarditis, cardiomyopathy, congenital heart diseases, or variant angina, and (3) those on oral antiarrhythmic drugs (classes I, III, and IV), those with persistent bundle branch block, or those with a complete pacemaker rhythm.

### 2.2 ECG grouping and diagnostic criteria

The first ECG after admission to the emergency department and before PCI was selected. The ECG was recorded with the standard 12 leads and the right precordial chest leads V_3_R–V_5_R and the posterior chest leas V_7_–V_9_ at a paper speed of 25 mm/s and an amplification of 10 mm/mV. Based on the R‐wave amplitude in lead V_1_ and the presence or absence of ST↑ in posterior leads, the patients were divided into four groups: Group A: R V_1_ <0.3 mV with ST↑ V_7_–V_9_, Group B: R V_1_ <0.3 mV without ST↑ V_7_–V_9_, Group C: R V_1_ ≥0.3 mV with ST↑ V_7_–V_9_, and Group D: R V_1_ ≥0.3 mV without ST↑ V_7_–V_9_ (Figure 1b). Among them, R V_1_ ≥0.3 mV in IMI represents lateral myocardial involvement (Goldwasser et al., 2015). The magnitude of ST‐segment deviation relative to the TP‐segment was measured at the J‐point in all leads. ST↑ >0.1 mV in two of the inferior leads was diagnosed as IMI. ST↑ >0.05 mV in leads V_3_R–V_5_R was diagnosed as RVMI, also the other signs for RVMI including QS or QR in leads V_3_R–V_5_R, QS in lead V_1_ and simultaneous ST↑ in lead V_1_, and depression in lead V_2_ were evaluated (Coma‐Canella et al., 1986; Mak et al., 1994; Morgera et al., 1984). The IBMI was diagnosed by ST↑ >0.05 mV in leads V_7_–V_9_. The diagnostic cut‐off value was based on the electrocardiographic content of the Fourth Universal Definition of Myocardial Infarction (Thygesen et al., 2018). The LV infarcted size, expressed as a percentage of LV mass, can be computed using the modified Aldrich score = 3 × (0.6 (∑ST↑ II, III, aVF) + 2.0) + 3 × (1.5 (number of other leads with ST↑) − 0.4) (Clemmensen et al., 1991). The early Q wave before reperfusion defined as Q duration ≥40 ms and depth ≥25% of the R and the Sclarovsky–Birnbaum Ischemia Grade was recorded in lead III (Topal et al., 2017). The Sclarovsky–Birnbaum Ischemia Grading System (SB‐IG) is an electrocardiographic method evaluating the severity of ischemia based on QRS‐ST‐T changes, but inverted or biphasic T is excluded (Billgren et al., 2004). Ventricular arrhythmias included frequent ventricular premature beats, paroxysmal ventricular tachycardia, and ventricular fibrillation.

### 2.3 Coronary angiography

Coronary artery stenosis was assessed by at least two experienced interventional cardiologists using multiangle projections from a multifunctional angiography catheter, accessed via the radial or femoral artery. The left main (LM) coronary artery or LAD artery disease was defined as >50% stenosis. Triple vessel disease was defined as >50% stenosis in the LAD artery, left circumflex (LCX) artery, and right coronary artery (RCA). Thrombolysis in myocardial infarction (TIMI) grades related to prognosis are defined as: 0, 1, 2, 3 = no, minimal, partial, normal flow respectively. TIMI 0–1 = effective occlusion (GUSTO Angiographic Investigators, 1993; Simes et al., 1995).

### 2.4 Statistical analysis

We employed IBM SPSS 22.0 for statistical analysis and data processing. Continuous variables were represented as mean ± standard deviation, and comparisons between groups were conducted using the *t*‐test. The times from symptom onset to ECG recording and angiography, as well as from door to wire through, were expressed as medians with interquartile ranges. Group comparisons were conducted using the Kruskal–Wallis test. Categorical data were presented as percentages or ratios, and comparisons were made using the chi‐squared test or Fisher's exact test. A *p*‐value less than .05 was deemed statistically significant.

## 3 RESULTS

### 3.1 Basic demographic characteristics, myocardial injury markers, and in‐hospital prognosis

**FIGURE 2:**
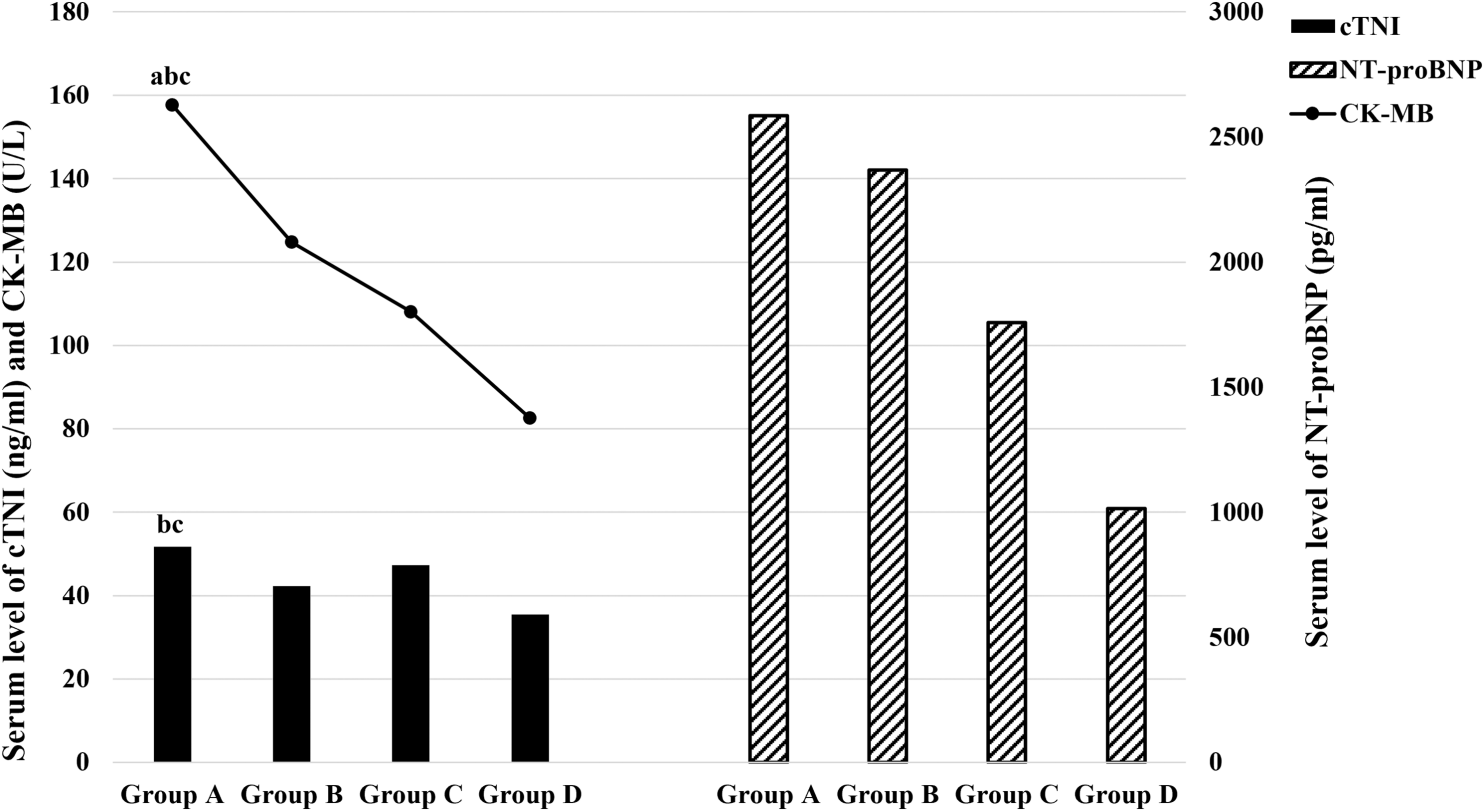
Comparisons of mean values of serum cTNI, CK‐MB, and NT‐proBNP among four groups. Group A: R V_1_ <0.3 mV with ST↑ V_7_–V_9_, Group B: R V_1_ <0.3 mV without ST↑ V_7_–V_9_, Group C: R V_1_ ≥0.3 mV with ST↑ V_7_–V_9_, Group D: R V_1_ ≥0.3 mV without ST↑ V_7_–V_9_. Compared with Group C, ^a^ *p* < .05, compared with Group D, ^b^ *p* < .05, compared with Group B, ^c^ *p* < .05. CK‐MB, creatine kinase MB isoform; cTNI, cardiac troponin I; NT‐proBNP, N‐terminal pro‐B‐type natriuretic peptide.

The age in Groups A and B was older than that in Group C, and glucose in Groups A and B was higher than that in the other two groups (*p* < .05). Group A had the highest creatine kinase MB isoform (CK‐MB) levels among four groups (*p* < .05). Both Groups A and C showed elevated cTNI compared to Groups B and D (*p* < .05 and *p* = .16, respectively). N‐terminal pro‐B‐type natriuretic peptide (NT‐proBNP) levels decreased sequentially across the groups (*p* = .20), with Group D having the highest left ventricular ejection fraction (LVEF) (*p* < .05) (Figure 2). There were no statistically significant differences in gender, disease spectrum, or serum biochemical parameters and arrhythmia, cardiogenic shock, or death among the four groups (all *p* > .05) (Table 1).

**TABLE 1:** Comparisons of clinical data, myocardial injury marker and in‐hospital prognosis among four groups ($\bar{x}$ ± s, medians and interquartile range, or %).

	Group A (*n* = 115)	Group B (*n* = 108)	Group C (*n* = 88)	Group D (*n* = 23)
Age (years)	62.83 ± 12.66^a^	62.54 ± 12.67^a^	58.53 ± 13.41	58.83 ± 12.95
Men (*n*, %)	86 (74.8)	83 (76.9)	76 (86.4)	22 (95.7)
Symptom to ECG recording (h)^†^	5.5 (2.0, 9.0)	6.5 (2.8, 9.0)	6.0 (3.3, 13.0)	6.0 (2.0, 12.5)
≤4 h (*n*, %)	48 (41.7)	49 (45.4)	39 (44.3)	8 (34.8)
4 to ≤12 h (*n*, %)	49 (42.6)	49 (45.4)	25 (28.4)	12 (52.2)
12 to ≤24 h (*n*, %)	18 (15.7)	10 (9.2)	24 (27.3)	3 (13.0)
Heart rate (beats/min)	74.40 ± 15.31	73.61 ± 16.99	77.31 ± 16.75	72.04 ± 14.18
Systolic blood pressure (mmHg)	123.34 ± 22.31	123.79 ± 27.37	118.48 ± 23.82	117.86 ± 22.73
Diastolic blood pressure (mmHg)	75.05 ± 16.09	74.13 ± 15.81	75.09 ± 15.40	74.71 ± 15.47
Smoking (*n*, %)	62 (53.9)	65 (60.2)	51 (58.0)	17 (73.9)
Drinking (*n*, %)*	16 (13.9)	18 (16.7)	14 (15.9)	5 (21.7)
Hypertension (*n*, %)	61 (53.0)	61 (56.5)	43 (48.9)	12 (52.2)
Diabetes (*n*, %)	28 (24.3)	26 (24.1)	17 (19.3)	4 (17.4)
Prior cerebral infarction (*n*, %)	22 (19.1)	13 (12.0)	10 (11.4)	1 (4.3)
Prior myocardial infarction (*n*, %)	13 (11.3)	9 (8.3)	11 (12.5)	0 (0)
Creatinine (μmol/L)	72.22 ± 27.45	70.99 ± 21.72	70.31 ± 14.70	71.72 ± 7.95
Sodium (mmol/L)	137.85 ± 3.77	136.49 ± 4.81	137.40 ± 3.92	137.98 ± 3.53
Potassium (mmol/L)	3.95 ± 0.46	3.96 ± 0.54	3.85 ± 0.44	4.12 ± 0.43
Glucose (mmol/L)	8.79 ± 3. 60^ab^	8.80 ± 3.97^ab^	7.58 ± 2.16	6.74 ± 2.26
CTNI (ng/mL)	51.77 ± 33.66^bc^	42.33 ± 26.28	47.36 ± 44.27	35.42 ± 17.01
CK‐MB (U/L)	157.62 ± 101.24^abc^	124.71 ± 95.92	108.06 ± 109.35	82.51 ± 73.69
NT‐proBNP (pg/mL)	2585.19 ± 1886.95	2367.51 ± 2431.51	1758.51 ± 2027.03	1013.94 ± 1430.30
LA (mm)	35.57 ± 5.47	34.67 ± 3.61	35.46 ± 4.71	34.82 ± 2.60
LV (mm)	47.66 ± 4.50	47.98 ± 4.32	48.15 ± 4.83	47.06 ± 4.51
LVEF (%)	52.02 ± 7.56^b^	52.68 ± 10.96^b^	53.38 ± 7.49^b^	57.90 ± 6.90
Killip classification				
І/II (*n*, %)	87 (75.7)	83 (76.9)	67 (76.1)	21 (91.3)
III/IV (*n*, %)	28 (24.3)	25 (23.1)	21 (23.9)	2 (8.7)
Sinus bradycardia or arrest (*n*, %)	36 (31.3)	35 (32.4)	26 (29.5)	5 (21.7)
Atrioventricular block (*n*, %)				
I°	20 (17.4)	17 (15.7)	10 (11.4)	2 (8.7)
II°	2 (1.7)	2 (1.9)	2 (2.3)	1 (4.3)
III°	7 (6.1)	5 (4.6)	1 (1.1)	0 (0)
Atrial premature or tachycardia (*n*, %)	1 (0.9)	2 (1.9)	1 (1.1)	1 (4.3)
Atrial fibrillation (*n*, %)	7 (6.1)	4 (3.7)	6 (6.8)	1 (4.3)
Ventricular arrhythmias (*n*, %)	18 (15.7)	10 (9.3)	9 (10.2)	1 (4.3)
Temporary pacemaker (*n*, %)	2 (1.7)	2 (1.9)	0 (0)	0 (0)
Cardiogenic shock (*n*, %)	16 (13.9)	18 (16.7)	13 (14.8)	2 (8.7)
Death (*n*, %)	7 (6.1)	1 (0.9)	4 (4.5)	0 (0)
Hospital stay (day)	9.00 ± 2.40	9.90 ± 4.16	9.25 ± 2.57	8.78 ± 2.95

*Note*: Group A: R V_1_ <0.3 mV with ST↑ V_7_–V_9_, Group B: R V_1_ <0.3 mV without ST↑ V_7_–V_9_, Group C: R V_1_ ≥0.3 mV with ST↑ V_7_–V_9_, Group D: R V_1_ ≥0.3 mV without ST↑ V_7_–V_9_. Compared with Group C, ^a^ *p* < .05, compared with Group D, ^b^ *p* < .05, compared with Group B, ^c^ *p* < .05.

Abbreviations: CK‐MB, creatine kinase MB isoform; cTNI, cardiac troponin I; LVEF, left ventricular ejection fraction; NT‐proBNP, N‐terminal pro‐B‐type natriuretic peptide.

* Drinking is defined as consuming eight standard drinks per week, each containing about 14 g of pure alcohol.

^†^ Detected by Kruskal–Wallis test.

### 3.2 Coronary angiographic findings

In Groups A and B, the culprit lesions were predominantly in the proximal or middle RCA. In Group C, they were frequently in the distal RCA or the proximal and middle LCX. In Group D, lesions were mainly in the distal segment of both the RCA and LCX (all *p* < .05). The initial TIMI blood flow grade, LAD disease, LM disease, and triple vessel disease showed no statistically significant difference across the four groups (Table 2).

**TABLE 2:** Comparisons of angiographic and procedural findings among four groups ($\bar{x}$ ± s, medians and interquartile range, or %).

	Group A (*n* = 115)	Group B (*n* = 108)	Group C (*n* = 88)	Group D (*n* = 23)
Symptom to angiography (h)^†^	8.4 (2.5, 11.4)	7.4 (3.4, 11.1)	6.9 (4.2, 11.7)	6.7 (2.6, 13.8)
Door to wire through (min)^†^	95.0 (70.0, 124.0)	67.5 (53.5, 101.0)	72.0 (59.0, 90.0)	60.0 (54.5, 118.5)
Initial PTCA (*n*, %)	13 (11.3)	10 (9.3)	24 (27.3)	0 (0)
Time of first PCI (min)	44.50 ± 17.77	36.44 ± 12.29	42.10 ± 15.45	45.78 ± 21.14
Initial stents deployed^‡^ ^,^ ^§^	1.30 ± 0.67	1.33 ± 0.49	1.80 ± 0.92	1.22 ± 0.44
Total hospital procedures	1.18 ± 0.39	1.32 ± 0.65	1.35 ± 0.49	1.33 ± 0.50
Total stents deployed^‡^	0.82 ± 0.81	1.45 ± 1.10	1.50 ± 0.97	1.67 ± 0.87
Culprit location (*n*, %)				
RCA				
Proximal	36 (31.3)^a^	38 (35.2)^a^	12 (13.6)	7 (30.4)
Middle	38 (33.0)^ab^	43 (39.8)^ab^	17 (19.3)	1 (4.3)
Distal	30 (26.1)	22 (20.4)	25 (28.4)	8 (34.8)
LCX				
Proximal	5 (4.3)^a^	1 (0.9)^a^	16 (18.2)	1 (4.3)
Middle	1 (0.9)^a^	(0)^ab^	9 (10.2)	2 (8.7)
Distal	5 (4.3)^b^	4 (3.7)^b^	9 (10.2)	4 (17.4)
Initial TIMI flow (*n*, %)				
0–1	85 (73.9)	79 (73.1)	64 (72.7)	18 (78.3)
2	10 (8.7)	14 (13.0)	9 (10.2)	3 (13.0)
3	20 (17.4)	15 (13.9)	15 (17.1)	2 (8.7)
LAD disease (*n*, %)	92 (80.0)	79 (73.1)	59 (67.0)	18 (78.3)
LM disease (*n*, %)	8 (7.0)	5 (4.6)	9 (10.2)	2 (8.7)
Triple vessel disease (*n*, %)	63 (54.8)	57 (52.8)	40 (45.5)	13 (56.5)

*Note*: Group A: R V_1_ <0.3 mV with ST↑ V_7_–V_9_, Group B: R V_1_ <0.3 mV without ST↑ V_7_–V_9_, Group C: R V_1_ ≥0.3 mV with ST↑ V_7_–V_9_, Group D: R V_1_ ≥0.3 mV without ST↑ V_7_–V_9_. Compared with Group C, ^a^ *p* < .05, compared with Group D, ^b^ *p* < .05.

Abbreviations: LAD, left descending coronary artery; LCX, left circumflex coronary artery; LM, left main coronary artery; PCI, percutaneous coronary intervention; PTCA, percutaneous transluminal coronary angioplasty; RCA, right coronary artery; TIMI, thrombolysis in myocardial infarction.

^†^ Detected by Kruskal–Wallis test.

^‡^ Drug balloon was included.

^§^ Only initial stenting patients were included.

### 3.3 ECG parameters

**FIGURE 3:**
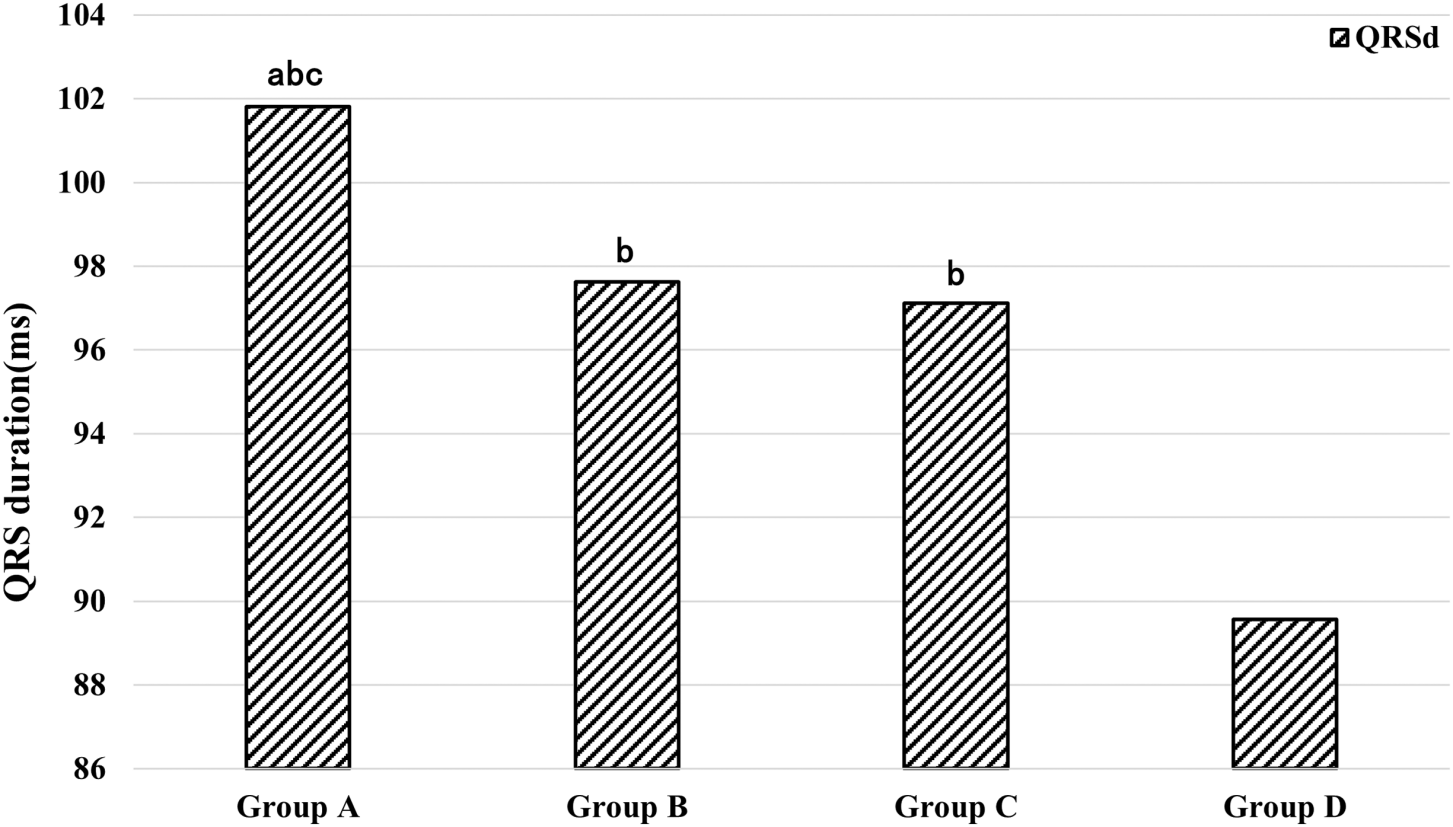
Comparisons of mean values of QRS duration (QRSd) among four groups. Group A: R V_1_ <0.3 mV with ST↑ V_7_–V_9_, Group B: R V_1_ <0.3 mV without ST↑ V_7_–V_9_, Group C: R V_1_ ≥0.3 mV with ST↑ V_7_–V_9_, Group D: R V_1_ ≥0.3 mV without ST↑ V_7_–V_9_. Compared with Group C, ^a^ *p* < .05, compared with Group D, ^b^ *p* < .05, compared with Group B, ^c^ *p* < .05.

**FIGURE 4:**
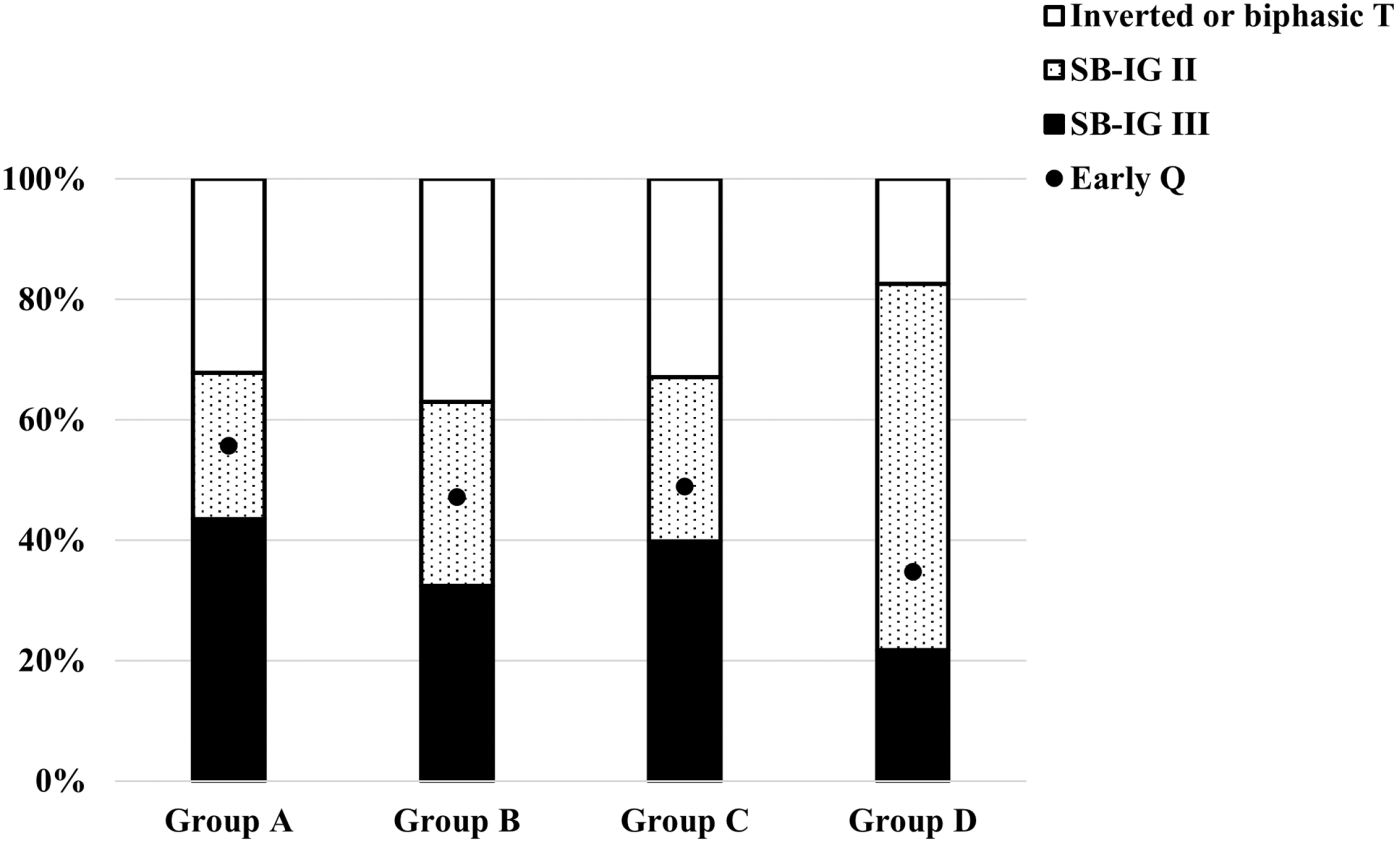
Comparisons of percent of SB‐IG, inverted or biphasic T and early Q among four groups. Group A: R V_1_ <0.3 mV with ST↑ V_7_–V_9_, Group B: R V_1_ <0.3 mV without ST↑ V_7_–V_9_, Group C: R V_1_ ≥0.3 mV with ST↑ V_7_–V_9_, Group D: R V_1_ ≥0.3 mV without ST↑ V_7_–V_9_. SB‐IG, Sclarovsky–Birnbaum Ischemia Grading System.

Our results showed that QRSd in Group A > Group B > Group C > Group D (*p* < .05) (Figure 3). Among the four groups, the proportion of SB‐IG II and Q wave was lowest in Group D (Figure 4). The proportions of RVMI indicators in Groups A and B were significantly higher than those in groups C and D (*p* < .01). IBMI was significantly associated with a greater sum of ST↑ in inferior leads and LV infarcted size. The extent of LV lateral injury is largest in Group C because of the highest ST↑ elevation in lead V_6_ (Table 3).

**TABLE 3:** Comparisons of ECG parameters among four groups ($\bar{x}$ ± s or %).

	Group A (*n* = 115)	Group B (*n* = 108)	Group C (*n* = 88)	Group D (*n* = 23)
QRS duration (ms)	101.81 ± 12.75^abc^	97.63 ± 14.69^b^	97.11 ± 11.84^b^	89.57 ± 9.79
QTc interval (ms)	422.10 ± 50.00	423.59 ± 53.75	411.66 ± 57.12	427.35 ± 24.13
PR interval (ms)	175.25 ± 40.19	169.10 ± 33.81	166.81 ± 31.90	169.59 ± 28.82
PJ interval (ms)	277.03 ± 43.23^ac^	265.30 ± 39. 58	262.61 ± 39.48	258.86 ± 36.09
Low voltage in limb leads (*n*, %)	22 (19.1)	22 (20.4)	19 (21.6)	4 (17.4)
Low voltage in all leads (*n*, %)	4 (3.5)	3 (2.8)	2 (2.3)	0 (0)
SB‐IG in lead III (*n*, %)				
II	28 (24.3)^b^	33 (30.6)^b^	24 (27.3)^b^	14 (60.9)
III	50 (43.5)	35 (32.4)	35 (39.8)	5 (21.7)
Inverted or biphasic T in III (*n*, %)	37 (32.2)	40 (37.0)	29 (33.0)	4 (17.4)
Early Q in III (*n*, %)	64 (55.7)	51 (47.2)	43 (48.9)	8 (34.8)
Signs for RVMI (*n*, %)				
ST↑ V_3_R	57 (49.6)^ab^	59 (54.6)^ab^	21 (23.9)	4 (17.4)
ST↑ V_4_R	76 (66.1)^ab^	79 (73.1)^ab^	25 (28.4)	5 (21.7)
ST↑ V_5_R	62 (53.9)^ab^	55 (50.9)^ab^	21 (23.9)	4 (17.4)
QS or QR in V_3_R	42 (36.5)^ab^	53 (49.1)^ab^	4 (4.5)	0 (0)
QS or QR in V_4_R	49 (42.6)^ab^	57 (52.8)^ab^	6 (6.8)	1 (4.3)
QS or QR in V_5_R	49 (42.6)^ab^	58 (53.7)^ab^	7 (8.0)	1 (4.3)
QS in V_1_	13 (11.3)^ac^	25 (23.1)^ab^	0 (0)	0 (0)
ST↑ V_1_ but ST↓ V_2_	31 (27.0)^a^	39 (36.1)^ab^	9 (10.2)	3 (13.0)
Any one of the above	92 (80.0)^ab^	88 (81.5)^ab^	27 (30.7)	5 (21.7)
ST↑ V_5_ and V_6_ (*n*, %)	44 (38.3)^abc^	3 (2.8%)	49 (55.7)^bc^	0 (0)
ST↑ V_6_ (mm)	0.23 ± 1.03^abc^	−0.75 ± 0.78^a^	0.56 ± 0.99^b^	−0.59 ± 0.63
Amplitude of R in V_1_ (mm)	1.03 ± 0.63^ab^	0.8 ± 0.57^ab^	3.82 ± 1.70	3.50 ± 1.48
R/S in V_1_	0.24 ± 0.34^ab^	0.18 ± 0.29^ab^	1.25 ± 1.27	1.52 ± 2.50
∑ST↑ II + III + aVF (mm)	4.89 ± 2.94^bc^	3.58 ± 2.21^a^	4.59 ± 2.93^b^	2.96 ± 1.47
∑ST↑ V_7_ + V_8_ + V_9_ (mm)	2.75 ± 1.80^bc^	−1.27 ± 1.62	3.13 ± 2.07^bc^	−0.44 ± 0.86
∑ST↑ V_3_R + V_4_R + V_5_R (mm)	1.78 ± 1.77^ab^	2.01 ± 1.61^ab^	0.07 ± 2.38	0.54 ± 1.34
LV infarcted size estimated by Aldrich score (%)*	18.81 ± 7.85^bc^	13.78 ± 5.04	19.22 ± 8.00^bc^	11.10 ± 3.30

*Note*: Group A: R V_1_ <0.3 mV with ST↑ V_7_–V_9_, Group B: R V_1_ <0.3 mV without ST↑ V_7_–V_9_, Group C: R V_1_ ≥0.3 mV with ST↑ V_7_–V_9_, Group D: R V_1_ ≥0.3 mV without ST↑ V_7_–V_9_. Compared with Group C, ^a^ *p* < .05, compared with Group D, ^b^ *p* < .05, compared with Group B, ^c^ *p* < .05.

Abbreviations: RVMI, right ventricular myocardial infarction; SB‐IG, Sclarovsky–Birnbaum Ischemia Grading System.

* LV infarcted size expressed by a percentage of the LV mass was calculated using the modified Aldrich score = 3 × (0.6 (∑ST↑ II, III, aVF) + 2.0) + 3 × (1.5 (no. of other leads with ST↑) − 0.4).

### 3.4 Stratifying findings in patients presenting <4 h

In 144 patients with inferior STEMI within 4 h, Group A showed the highest cTNI, CK‐MB, and NT‐proBNP, and the lowest LVEF, with significant differences only versus Group D (*p* < .05). Group A also had the most patients with SB‐IG III among all groups (*p* < .05) (Table 4).

**TABLE 4:** Comparisons of clinical data and ECG parameters among four groups in patients presenting <4 h ($\bar{x}$ ± s or %).

	Group A (*n* = 48)	Group B (*n* = 49)	Group C (*n* = 39)	Group D (*n* = 8)
CTNI (ng/mL)	68.50 ± 19.72^b^	58.92 ± 24.0	60.95 ± 27.86	40.50 ± 12.44
CK‐MB (U/L)	135.97 ± 60.09^b^	100.81 ± 50.70	114.27 ± 61.61	85.13 ± 52.63
NT‐proBNP (pg/mL)	2707.50 ± 1654.43^b^	624.60 ± 631.99	1471.83 ± 1080.56	330.67 ± 258.53
LVEF (%)	50.00 ± 5.80^b^	54.40 ± 9.09^b^	54.25 ± 7.25^b^	55.67 ± 4.62
QRS duration (ms)	104.75 ± 12.98^b^	102.30 ± 15.45	106.38 ± 9.20^b^	91.33 ± 2.31
SB‐IG in lead III (*n*, %)				
II	6 (12.5)^b^	29 (59.2)	20 (51.3)	6 (75)
III	42 (87.5)^abc^	15 (30.6)	9 (23.1)	2 (25)
Inverted or biphasic T in III (*n*, %)	0 (0)^a^	5 (10.2)	10 (25.6)	0 (0)
Early Q in III (*n*, %)	18 (37.5)^b^	15 (30.6)	15 (38.5)^b^	0 (0)
Signs for RVMI (*n*, %)	30 (62.5)^ab^	29 (59.2)^a^	5 (12.8)	2 (25)
LV infarcted size estimated by Aldrich score (%)*	23.72 ± 6.90^bc^	16.82 ± 6.56	22.86 ± 8.92^bc^	13.60 ± 6.21

*Note*: Group A: R V_1_ <0.3 mV with ST↑ V_7_–V_9_, Group B: R V_1_ <0.3 mV without ST↑ V_7_–V_9_, Group C: R V_1_ ≥0.3 mV with ST↑ V_7_–V_9_, Group D: R V_1_ ≥0.3 mV without ST↑ V_7_–V_9_. Compared with Group C, ^a^ *p* < .05, compared with Group D, ^b^ *p* < .05, compared with Group B, ^c^ *p* < .05.

Abbreviations: RVMI, right ventricular myocardial infarction; SB‐IG, Sclarovsky–Birnbaum Ischemia Grading System.

* LV infarcted size expressed by a percentage of the LV mass was calculated using the modified Aldrich score = 3 × (0.6 (∑ST↑ II, III, aVF) + 2.0) + 3 × (1.5 (no. of other leads with ST↑) − 0.4).

## 4 DISCUSSION

Similar to the significance of the early “pathological Q wave” in STEMI, the “pathological R wave” in lead V_1_ presumably represent severe lateral injury in inferior STEMI's acute phase, but the R‐wave amplitude can be offset by right ventricular infarction. Our study highlights a patient subgroup with larger infarct sizes, marked by an R wave <0.3 mV in lead V_1_ and ST↑ in leads V_7_–V_9_.

### 4.1 QRS wave morphology in lead V_1_ is affected by multiple infarct regions

Approximately two‐thirds of patients with inferior myocardial infarction (IMI) present with a lateral wall myocardial infarction. The QRS waveform change in lead V_1_ can be influenced by myocardial necrosis in different regions. (1) de Luna et al. revealed that the electrocardiographic activity of the lateral wall can be represented in the right thoracic V_1_ lead. In their study, which involved 45 patients with IMI confirmed by cardiac magnetic resonance imaging (CMRI), 23 displayed R V_1_ >3 mm, and 22 had evidence of lateral wall myocardial necrosis. The sensitivity, specificity, and negative predictive value of R V_1_ >3 mm in predicting lateral myocardial infarction stood at 73.3%, 93.3%, and 63.6% respectively (de Luna et al., 2008). (2) However, it is crucial to note that ECG changes in the right thoracic leads are influenced by the combined electrical activities of both the RV and the lateral wall of left ventricle (LV) (Lew et al., 1985; Mukharji et al., 1984). Roeslerb et al. conducted autopsies on five IMI patients exhibiting reduced Q‐ or R‐wave amplitude in right precordial leads. Their findings revealed interventricular septal necrosis extending from the anterior to the posterior regions. In some instances, there was concurrent myocardial necrosis in the adjacent septum (Roesler & Dressler, 1947). Subsequently, Halkett and Khan documented six IMI patients with ST↑ in leads V_1_–V_3_/V_4_ and a QS or rS waveform. Ventriculography or cardiac color Doppler ultrasound highlighted RV dilatation or diminished RV functionality. Meanwhile, the LV wall and interventricular septum remained functional. Coronary angiography affirmed RCA occlusion, yet the LAD artery remained patent. Their conclusion was clear: the necrotic Q wave in leads V_1_–V_3_ was not indicative of anterior septal or anterior wall myocardial infarction but was a manifestation of RV infarction (Halkett et al., 1986; Khan & Chou, 1996). (3) Advances in cardiac imaging have shown that leads V_1_–V_3_ are associated with the heart's inferior (posterior) septal segment. A Q wave in V_1_ signifies myocardial perfusion defects in the infero‐septum of LV basal segment (Jia et al., 2018; Zafrir et al., 2004).

### 4.2 Differential diagnosis of QS wave in lead V_1_

QS waves in lead V_1_ are commonly observed in various scenarios: septal myocardial scarring of nonischemic etiology (Ghadban et al., 2018); isolated septal myocardial infarction (Harimoto et al., 2019; Tomcsanyi et al., 2012); and anterior or apical myocardial infarction (Allencherril et al., 2018; Bogaty et al., 2002). It is rarely that a decrease in R‐wave amplitude or the presence of Q waves in lead V_1_ is attributed clinically to RV myocardial injury. However, theoretically, this association is feasible, as supported by autopsy findings (Lopez‐Sendon et al., 1985). Some cases have also reported isolated RV or RV outflow tract infarctions, visible on CMRI, being attributed to nondominant RCA or conal branch occlusion. These events can reduce the R‐wave amplitude in lead V_1_ and even produce pathological Q waves in leads V_2_ and V_3_ (Halkett et al., 1986; Khan & Chou, 1996; Lyle et al., 2016; Zhong et al., 2019). Furthermore, a Qr or QR morphology in lead V_1_ during acute pulmonary embolism is linked with increased RV stress (Kucher et al., 2003). This is attributed to RV myocardial injury and is seen as an indicator of RV insufficiency. Notably, such patients have a 3.62‐fold increased risk of cardiogenic shock (Kukla et al., 2014). All these findings may lend support to our speculation.

### 4.3 Probable mechanism decreasing R wave of lead V_1_ in inferior myocardial infarction

Consequently, the R‐wave amplitude in lead V_1_ is influenced by myocardial infarction in both the LV lateral wall and the RV, among other sites. During an episode of IMI, the diminished R wave, or the shift to rS or even QS patterns in lead V_1_, is presumably due to a deficiency in the normal left‐to‐right depolarization vector caused by the RV free wall. Even when the lateral wall is involved, R V_1_ >0.3 mV may not appear on the ECG at this juncture. This is because the right‐to‐left infarction force from the RV is predominant, which counterbalances the left‐to‐right infarction vector from the LV lateral wall. Consequently, the composite vector deviates from lead V_1_, diminishing the R‐wave amplitude in this lead. We postulate that this hypothesis explains the reduced sensitivity and negative predictive value of R V1 >0.3 mV in diagnosing lateral wall involvement, particularly when encompassing acute‐phase IMI patients and expanding the sample size (45 vs. 155 patients with inferior and lateral wall myocardial infarction, the sensitivity and negative predictive values were 73.4% vs. 27.7%, and 63.6% vs. 41.6%, respectively) (Goldwasser et al., 2015). Additionally, the incidence of the proximal RCA being the culprit artery was higher in patients in Groups A and B than in Groups C and D. The obstruction of this proximal segment diminishes the blood supply from the posterior descending artery to the lower (posterior) septal. The resultant ischemia further weakens the R wave in lead V_1_.

The ST↑ magnitude correlates with the extent of myocardial injury (Hathaway et al., 1998). Studies have indicated that the sum of absolute ST‐segment deviations can predict patient prognosis. Based on this, in our study, we hypothesized that the severity of myocardial injury or infarction in STEMI patients can be partly inferred from the acute ∑ST↑ in ECGs recorded before reperfusion therapy. We interpret ∑ST↑ II + III + aVF, ∑ST↑ V_7_ + V_8_ + V_9_, and ∑ST↑ V_3_R + V_4_R + V_5_R as indications of inferior, inferobasal, and right wall myocardial injury or infarction, respectively. Additionally, ST↑ in V_6_ indicates lateral involvement in the acute phase of inferior STEMI (Norda et al., 2015). We, therefore, consider ST↑ V_6_ as a marker of lateral injury extent. Notably, patients in Group C exhibit a larger lateral injury extent than those in Group A, yet the LV infarcted sizes, estimated by the modified Aldrich score, are comparable between both groups. However, the total infarcted extent, as estimated by serum markers, is higher in Group A. This discrepancy suggests that the more extensive myocardial injury in Group A may result from a significant right ventricular involvement, as evidenced by the higher ∑ST↑ V_3_R + V_4_R + V_5_R in Group A compared to Group C. The explanation is particularly relevant for inferior STEMI >4 h, given that the necrotic extent, estimated by CK‐MB or cTNI, is similar in those <4 h in both Groups A and C. However, in early‐onset patients (<4 h) with additional ST elevation in leads V_7_–V_9_, the R V_1_ <3 mm suggests worse left heart function. This is indicated by higher NT‐proBNP (*p* = .377) and lower LVEF (*p* = .286) in Group A, likely due to RVMI impairing left ventricular filling (Nägele & Flammer, 2022) and more limited collateral circulation in the LV itself, evidenced by the fact that more patients in Group A presented with severe ischemia (SB‐IG III) (Billgren et al., 2004).

### 4.4 ST↑ in leads V_7_–V_9_ denotes a more severe ischemic event than its absence in inferior myocardial infarction

Matetzky S et al. demonstrated that ST↑ in leads V_7_–V_9_ signifies abnormalities in the motion of the inferior or inferolateral wall of the basal segment, as evidenced by echocardiography. Therefore, in our study, the term “inferobasal myocardial infarction (IBMI)” is used to denote the ST↑ in V_7_–V_9_, a terminology that aligns with the fourth definition of myocardial infarction. Roughly 53% of patients with IMI present with IBMI, with the responsible artery being either the RCA, LCX, or their branches. It is noteworthy that patients diagnosed with both IMI and IBMI exhibit a heightened incidence of congestive heart failure, reinfarction, mortality, and mitral regurgitation compared to those without IBMI. The extent of the infarct in such cases is comparable to that observed in anterior MI. Additionally, these patients are more likely to derive significant benefits from revascularization therapy (Adawi & Atar, 2008; Matetzky et al., 1998). In our Groups A and C, patients with concurrent IBMI exhibited higher cTNI levels compared to Groups B and D without IBMI. Group D patients demonstrated a superior LVEF, suggesting a more extensive myocardial damage and compromised LV systolic function when paired with IBMI. Although Groups A and B saw a heightened prevalence of cardiogenic shock, there was not a significant statistical difference, as well as in NT‐proBNP trends among the groups. This could be attributed to the mitigative effects of timely PCI in preventing complications for some patients.

### 4.5 Implications of prolonged QRS duration in myocardial infarction

Our study indicated a progressive shortening of the QRSd from Group A to D, with the longest duration observed when R V_1_ <0.3 mV was combined with IBMI. We speculate this is due to: (1) under hypoxia and acidosis with sodium–potassium pump inhibition or potassium ATP channel activation, localized hyperkalemia slows conduction in ischemic areas. The larger the ischemic area, as seen in Group A patients, often with concurrent RV and inferobasal ischemia, the longer the QRSd (Hoeker et al., 2020; Terkildsen et al., 2007). (2) Ischemia extending to the LV's posterobasal wall and the RV's basal segment delays the ventricular depolarization (Durrer et al., 1970), especially the former is prominent in the hyperacute stage (<4 h), as equally longer QRSd in both Groups A and C with ST elevation in posterior leads. However, when including patients presenting between 4 and 24 h, only Group A exhibited the longest QRSd, whereas Group C experienced earlier QRSd shortening. We consider that ongoing RV ischemia plays a significant role in the later stages (>4 h). The variation in QRSd over different time is hypothesized to the distinct ischemic tolerances and metabolic reactions of the left and right ventricles (Nägele & Flammer, 2022). (3) Cardiac insufficiency, paired with compensatory cardiomyocyte and interstitial proliferation, increases QRSd due to heightened electrical conduction resistance (García‐Escobar et al., 2022). In non‐STEMI patients, Shah M et al. identified that a QRSd ≥90 ms indicates significant coronary artery involvement (Shah et al., 2016). Some studies correlate QRSd between 90 and 120 ms with increased cardiac volumes, reduced LVEF, and in‐hospital cardiovascular events (Taskesen et al., 2014). Yusuf J et al. further related longer QRSd with impaired microvascular reperfusion in STEMI patients (Yusuf et al., 2018). Our results underscore the significance of QRSd in assessing AMI patient prognosis.

## 5 LIMITATIONS

There are some limitations to this study. Firstly, the sample size of “R V1 ≥0.3 mV without IBMI” in Group D was too small, probably because the pathological R wave in lead V_1_ predict the “(aterolateral) lateral wall” injury, and the ST↑ in the V_7_–V_9_ leads represents inferior basal or partly the posterior lateral wall (Sclarovsky et al., 1987), above infarcted positions are adjacent, hence the coexistence of those is common. Secondly, complete ST‐segment resolution post‐PCI indicates successful reperfusion, while Q‐wave regression implies enhanced LV function. However, the lack of postreperfusion ST‐segment recovery and ECG follow‐up, their prognostic value in our groups is undetermined. Thirdly, approximately 18% of our patients were treated after 12 h, missing the optimal reperfusion window, and the fact that only 43.1% arrived within 4 h limits comparison with other studies such as Andrews et al. included only patients presenting at <4 h. Yet, despite the time‐related variability in myocardial infarction severity, our findings provide a potential explanation. Lastly, post‐PCI, our center enforces a 3‐month waiting period before conducting CMRI to mitigate stent‐related concerns, despite evidence support that early CMRI in AMI patients is safe and not posing potential risks. Hence, patients also did not undergo CMRI to clarify the specific infarct site or area at risk in the acute phase. Large‐scale studies combining the imaging evaluation and time‐dependent ECG stratification are needed to further test our proposition.

## 6 CONCLUSION

We identified a high‐risk subset of patients with inferior myocardial infarction, characterized by a decreased R‐wave amplitude in lead V_1_ and ST↑ in leads V_7_–V_9_. A potential underlying mechanism is the greater infarct size in the right ventricle compared to the lateral wall of the left ventricle, with the inferobasal wall area also affected. Such patients exhibit prolonged QRS duration, most indicative of compromised cardiac function within 4 h post‐inferior STEMI, yet they benefit significantly from interventional treatments in the widespread adoption of PCI.

## AUTHOR CONTRIBUTIONS

Xiao‐Bin Zheng designed the study, performed data analysis, and wrote the manuscript. Hai‐Yan Wu created the figures. Ming Zhang collected and analyzed ECG data. Bing‐Qi Yao collected clinical data. All authors reviewed and approved the final manuscript.

## CONFLICT OF INTEREST STATEMENT

None.

## ETHICS STATEMENT

The study conforms to the Helsinki Declaration and has been approved by the Hospital Ethics Committee.

## PATIENT CONSENT STATEMENT

The study was done after agreement from the Hospital Ethics Committee and with the patients' informed consent.

## PERMISSION TO REPRODUCE MATERIAL FROM OTHER SOURCES

We have not reproduced material from other sources.

## CLINICAL TRIAL REGISTRATION

Because our study was retrospective, we did not registered the trial on clinicaltrials.gov at the beginning.

## Data Availability

Due to privacy issues, these data are not publicly available.

## References

[anec13114-bib-0001] Adawi, K. , & Atar, S. (2008). Clinical implications and angiographic and electrocardiographic correlation of ST segment elevation in leads V7‐V9 in patients with ST elevation myocardial infarction. Harefuah, 147(7), 587–590. 18814514

[anec13114-bib-0002] Allencherril, J. , Fakhri, Y. , Engblom, H. , Heiberg, E. , Carlsson, M. , Dubois‐Rande, J. L. , Halvorsen, S. , Hall, T. S. , Larsen, A. I. , Jensen, S. E. , Arheden, H. , Atar, D. , Clemmensen, P. , Shah, D. J. , Cheong, B. , Sejersten, M. , & Birnbaum, Y. (2018). Appropriateness of anteroseptal myocardial infarction nomenclature evaluated by late gadolinium enhancement cardiovascular magnetic resonance imaging. Journal of Electrocardiology, 51(2), 218–223. 10.1016/j.jelectrocard.2017.09.013 29103621

[anec13114-bib-0003] Andrews, J. , French, J. K. , Manda, S. O. , & White, H. D. (2000). New Q waves on the presenting electrocardiogram independently predict increased cardiac mortality following a first ST‐elevation myocardial infarction. European Heart Journal, 21(8), 647–653. 10.1053/euhj.1999.1908 10731402

[anec13114-bib-0004] Armstrong, P. W. , Fu, Y. , Westerhout, C. M. , Hudson, M. P. , Mahaffey, K. W. , White, H. D. , Todaro, T. G. , Adams, P. X. , Aylward, P. E. , & Granger, C. B. (2009). Baseline Q‐wave surpasses time from symptom onset as a prognostic marker in ST‐segment elevation myocardial infarction patients treated with primary percutaneous coronary intervention. Journal of the American College of Cardiology, 53(17), 1503–1509. 10.1016/j.jacc.2009.01.046 19389560

[anec13114-bib-0005] Bateman, T. M. , Czer, L. S. , Gray, R. J. , Maddahi, J. , Raymond, M. J. , Geft, I. L. , Ganz, W. , Shah, P. K. , & Berman, D. S. (1983). Transient pathologic Q waves during acute ischemic events: An electrocardiographic correlate of stunned but viable myocardium. American Heart Journal, 106(6), 1421–1426. 10.1016/0002-8703(83)90056-x 6650366

[anec13114-bib-0006] Bayés de Luna, A. , Rovai, D. , Pons Llado, G. , Gorgels, A. , Carreras, F. , Goldwasser, D. , & Kim, R. J. (2015). The end of an electrocardiographic dogma: A prominent R wave in V1 is caused by a lateral not posterior myocardial infarction‐new evidence based on contrast‐enhanced cardiac magnetic resonance‐electrocardiogram correlations. European Heart Journal, 36(16), 959–964. 10.1093/eurheartj/ehv035 25666323

[anec13114-bib-0007] Billgren, T. , Birnbaum, Y. , Sgarbossa, E. B. , Sejersten, M. , Hill, N. E. , Engblom, H. , … Wagner, G. S. (2004). Refinement and interobserver agreement for the electrocardiographic Sclarovsky‐Birnbaum ischemia grading system. Journal of Electrocardiology, 37(3), 149–156. 10.1016/j.jelectrocard.2004.02.005 15286927

[anec13114-bib-0008] Bogaty, P. , Boyer, L. , Rousseau, L. , & Arsenault, M. (2002). Is anteroseptal myocardial infarction an appropriate term? The American Journal of Medicine, 113(1), 37–41. 10.1016/s0002-9343(02)01050-1 12106621

[anec13114-bib-0009] Clemmensen, P. , Grande, P. , Aldrich, H. R. , & Wagner, G. S. (1991). Evaluation of formulas for estimating the final size of acute myocardial infarcts from quantitative ST‐segment elevation on the initial standard 12‐lead ECG. Journal of Electrocardiology, 24(1), 77–83. 10.1016/0022-0736(91)90084-y 2056271

[anec13114-bib-0010] Coma‐Canella, I. , López‐Sendón, J. , Alcasena, S. , García, C. , Gamallo, C. , & Jadraque, L. M. (1986). Electrocardiographic alterations in leads V1 to V3 in the diagnosis of right and left ventricular infarction. American Heart Journal, 112(5), 940–946. 10.1016/0002-8703(86)90304-2 3776820

[anec13114-bib-0011] de Luna, A. B. , Cino, J. , Goldwasser, D. , Kotzeva, A. , Elosua, R. , Carreras, F. , Pujadas, S. , Garcia‐Moll, X. , Santaló, M. , Fiol, M. , Bayés‐Genís, A. , Pons‐Lladó, G. , & Cinca, J. (2008). New electrocardiographic diagnostic criteria for the pathologic R waves in leads V1 and V2 of anatomically lateral myocardial infarction. Journal of Electrocardiology, 41(5), 413–418. 10.1016/j.jelectrocard.2007.10.002 18721647

[anec13114-bib-0012] Durrer, D. , van Dam, R. T. , Freud, G. E. , Janse, M. J. , Meijler, F. L. , & Arzbaecher, R. C. (1970). Total excitation of the isolated human heart. Circulation, 41(6), 899–912. 10.1161/01.cir.41.6.899 5482907

[anec13114-bib-0013] García‐Escobar, A. , Vera‐Vera, S. , Jurado‐Román, A. , Jiménez‐Valero, S. , Galeote, G. , & Moreno, R. (2022). Subtle QRS changes are associated with reduced ejection fraction, diastolic dysfunction, and heart failure development and therapy responsiveness: Applications for artificial intelligence to ECG. Annals of Noninvasive Electrocardiology, 27(6), e12998. 10.1111/anec.12998 35904538 PMC9674781

[anec13114-bib-0014] Ghadban, R. , Alpert, M. A. , Dohrmann, M. L. , Allaham, H. , Payne, J. E. , Fong, H. K. , & Kumar, S. A. (2018). A QS pattern in leads V_1_ and V_2_ is associated with septal scarring independent of scar etiology – A cardiac magnetic resonance imaging study. Journal of Electrocardiology, 51(4), 577–582. 10.1016/j.jelectrocard.2018.03.011 29996993

[anec13114-bib-0015] Goldwasser, D. , Senthilkumar, A. , Bayés de Luna, A. , Elosua, R. , Carreras, F. , Pons‐Llado, G. , & Kim, R. J. (2015). Lateral MI explains the presence of prominent R wave (R ≥ S) in V1. Annals of Noninvasive Electrocardiology, 20(6), 570–577. 10.1111/anec.12260 25764092 PMC6931754

[anec13114-bib-0016] Gupta, T. , Weinreich, M. , Kolte, D. , Khera, S. , Villablanca, P. A. , Bortnick, A. E. , Wiley, J. M. , Menegus, M. A. , Kirtane, A. J. , Bhatt, D. L. , Garcia, M. J. , Latib, A. , & Weisz, G. (2020). Comparison of incidence and outcomes of cardiogenic shock complicating posterior (inferior) versus anterior ST‐elevation myocardial infarction. The American Journal of Cardiology, 125(7), 1013–1019. 10.1016/j.amjcard.2019.12.052 31955831

[anec13114-bib-0017] GUSTO Angiographic Investigators . (1993). The effects of tissue plasminogen activator, streptokinase, or both on coronary‐artery patency, ventricular function, and survival after acute myocardial infarction. The New England Journal of Medicine, 329(22), 1615–1622. 10.1056/NEJM199311253292204 8232430

[anec13114-bib-0018] Halkett, J. A. , Commerford, P. J. , & Millar, R. S. (1986). Right ventricular infarction mimicking extensive anterior infarction. Chest, 90(4), 617–619. 10.1378/chest.90.4.617 3757576

[anec13114-bib-0019] Harimoto, K. , Kawasaki, T. , Honda, S. , Kinoshita, S. , Kamitani, T. , & Sugihara, H. (2019). A case of isolated septal myocardial infarction: Myocardial perfusion‐metabolism mismatch as a tool for diagnosis. Oman Medical Journal, 34(3), 257–261. 10.5001/omj.2019.49 31110636 PMC6505347

[anec13114-bib-0020] Hathaway, W. R. , Peterson, E. D. , Wagner, G. S. , Granger, C. B. , Zabel, K. M. , Pieper, K. S. , Clark, K. A. , Woodlief, L. H. , & Califf, R. M. (1998). Prognostic significance of the initial electrocardiogram in patients with acute myocardial infarction. GUSTO‐I investigators. Global utilization of streptokinase and t‐PA for occluded coronary arteries. JAMA, 279(5), 387–391. 10.1001/jama.279.5.387 9459474

[anec13114-bib-0021] Hoeker, G. S. , James, C. C. , Tegge, A. N. , Gourdie, R. G. , Smyth, J. W. , & Poelzing, S. (2020). Attenuating loss of cardiac conduction during no‐flow ischemia through changes in perfusate sodium and calcium. American Journal of Physiology. Heart and Circulatory Physiology, 319(2), H396–H409. 10.1152/ajpheart.00112.2020 32678707 PMC7473923

[anec13114-bib-0022] Jia, X. , Heiberg, E. , Sejersten Ripa, M. , Engblom, H. , Carlsson, M. , Halvorsen, S. , Arheden, H. , Atar, D. , Clemmensen, P. , & Birnbaum, Y. (2018). Cardiac magnetic resonance evaluation of the extent of myocardial injury in patients with inferior ST elevation myocardial infarction and concomitant ST depression in leads V1‐V3: Analysis from the MITOCARE study. Cardiology, 140(3), 178–185. 10.1159/000491745 30099440

[anec13114-bib-0023] Khan, Z. U. , & Chou, T. C. (1996). Right ventricular infarction mimicking acute anteroseptal left ventricular infarction. American Heart Journal, 132(5), 1089–1093. 10.1016/s0002-8703(96)90038-1 8892800

[anec13114-bib-0024] Kucher, N. , Walpoth, N. , Wustmann, K. , Noveanu, M. , & Gertsch, M. (2003). QR in V1 – An ECG sign associated with right ventricular strain and adverse clinical outcome in pulmonary embolism. European Heart Journal, 24(12), 1113–1119. 10.1016/s0195-668x(03)00132-5 12804925

[anec13114-bib-0025] Kukla, P. , McIntyre, W. F. , Fijorek, K. , Mirek‐Bryniarska, E. , Bryniarski, L. , Krupa, E. , Jastrzębski, M. , Bryniarski, K. L. , Zhong‐qun, Z. , & Baranchuk, A. (2014). Electrocardiographic abnormalities in patients with acute pulmonary embolism complicated by cardiogenic shock. The American Journal of Emergency Medicine, 32(6), 507–510. 10.1016/j.ajem.2014.01.043 24602894

[anec13114-bib-0026] Lawton, J. S. , Tamis‐Holland, J. E. , Bangalore, S. , Bates, E. R. , Beckie, T. M. , Bischoff, J. M. , Bittl, J. A. , Cohen, M. G. , DiMaio, J. M. , Don, C. W. , Fremes, S. E. , Gaudino, M. F. , Goldberger, Z. D. , Grant, M. C. , Jaswal, J. B. , Kurlansky, P. A. , Mehran, R. , Metkus, T. S., Jr. , Nnacheta, L. C. , … Zwischenberger, B. A. (2022). 2021 ACC/AHA/SCAI guideline for coronary artery revascularization: A report of the American College of Cardiology/American Heart Association joint committee on clinical practice guidelines. Circulation, 145(3), e18–e114. 10.1161/CIR.0000000000001038 34882435

[anec13114-bib-0027] Lew, A. S. , Maddahi, J. , Shah, P. K. , Weiss, A. T. , Peter, T. , Berman, D. S. , & Ganz, W. (1985). Factors that determine the direction and magnitude of precordial ST‐segment deviations during Inferior Wall acute myocardial infarction. The American Journal of Cardiology, 55(8), 883–888. 10.1016/0002-9149(85)90711-8 3984877

[anec13114-bib-0028] Lopez‐Sendon, J. , Coma‐Canella, I. , Alcasena, S. , Seoane, J. , & Gamallo, C. (1985). Electrocardiographic findings in acute right ventricular infarction: Sensitivity and specificity of electrocardiographic alterations in right precordial leads V4R, V3R, V1, V2, and V3. Journal of the American College of Cardiology, 6(6), 1273–1279. 10.1016/s0735-1097(85)80213-8 4067105

[anec13114-bib-0029] Lyle, M. , Van Woerkom, R. C. , Tweet, M. , Young, P. M. , & Best, P. J. (2016). Conus artery occlusion causing isolated right ventricular outflow tract infarction: Novel application of cardiac magnetic resonance in anterior STEMI. Cardiovascular Diagnosis and Therapy, 6(3), 262–266. 10.21037/cdt.2015.11.05 27280090 PMC4880749

[anec13114-bib-0030] Mak, K. H. , Chia, B. L. , Tan, A. T. , & Johan, A. (1994). Simultaneous ST‐segment elevation in lead V1 and depression in lead V2. A discordant ECG pattern indicating right ventricular infarction. Journal of Electrocardiology, 27(3), 203–207. 10.1016/s0022-0736(94)80003-0 7930982

[anec13114-bib-0031] Matetzky, S. , Freimark, D. , Chouraqui, P. , Rabinowitz, B. , Rath, S. , Kaplinsky, E. , & Hod, H. (1998). Significance of ST segment elevations in posterior chest leads (V7 to V9) in patients with acute inferior myocardial infarction: Application for thrombolytic therapy. Journal of the American College of Cardiology, 31(3), 506–511. 10.1016/s0735-1097(97)00538-x 9502627

[anec13114-bib-0032] Morgera, T. , Alberti, E. , Silvestri, F. , Pandullo, C. , Della Mea, M. T. , & Camerini, F. (1984). Right precordial ST and QRS changes in the diagnosis of right ventricular infarction. American Heart Journal, 108(1), 13–18. 10.1016/0002-8703(84)90538-6 6731262

[anec13114-bib-0033] Mukharji, J. , Murray, S. , Lewis, S. E. , Croft, C. H. , Corbett, J. R. , Willerson, J. T. , & Rude, R. E. (1984). Is anterior ST depression with acute transmural inferior infarction due to posterior infarction? A vectorcardiographic and scintigraphic study. Journal of the American College of Cardiology, 4(1), 28–34. 10.1016/s0735-1097(84)80314-9 6330194

[anec13114-bib-0034] Nägele, M. P. , & Flammer, A. J. (2022). Heart failure after right ventricular myocardial infarction. Current Heart Failure Reports, 19(6), 375–385. 10.1007/s11897-022-00577-8 36197627 PMC9653315

[anec13114-bib-0035] Norda, S. , van der Weg, K. , Vos, R. , & Gorgels, A. P. (2015). Electrocardiographic prediction of lateral involvement in acute non‐anterior wall myocardial infarction. Journal of Electrocardiology, 48(4), 527–532. 10.1016/j.jelectrocard.2015.02.005 25766970

[anec13114-bib-0036] O'Gara, P. T. , Kushner, F. G. , Ascheim, D. D. , Casey, D. E., Jr. , Chung, M. K. , de Lemos, J. A. , Ettinger, S. M. , Fang, J. C. , Fesmire, F. M. , Franklin, B. A. , Granger, C. B. , Krumholz, H. M. , Linderbaum, J. A. , Morrow, D. A. , Newby, L. K. , Ornato, J. P. , Ou, N. , Radford, M. J. , Tamis‐Holland, J. E. , … American College of Cardiology Foundation/American Heart Association Task Force on Practice Guidelines . (2013). 2013 ACCF/AHA guideline for the management of ST‐elevation myocardial infarction: A report of the American College of Cardiology Foundation/American Heart Association task force on practice guidelines. Circulation, 127(4), e362–e425. 10.1161/CIR.0b013e3182742cf6 23247304

[anec13114-bib-0037] Raitt, M. H. , Maynard, C. , Wagner, G. S. , Cerqueira, M. D. , Selvester, R. H. , & Weaver, W. D. (1995). Appearance of abnormal Q waves early in the course of acute myocardial infarction: Implications for efficacy of thrombolytic therapy. Journal of the American College of Cardiology, 25(5), 1084–1088. 10.1016/0735-1097(94)00514-q 7897120

[anec13114-bib-0038] Rijnierse, M. T. , Verouden, N. J. , & de Winter, R. J. (2012). Precordial R‐wave reappearance predicting infarct size and myocardial recovery after acute STEMI. Netherlands Heart Journal, 20(7–8), 326–329. 10.1007/s12471-011-0162-9 21611849 PMC3402577

[anec13114-bib-0039] Roesler, H. , & Dressler, W. (1947). An electrocardiographic pattern of infarction of the interventricular septum, extending from the anterior to the posterior aspect of the heart. American Heart Journal, 34(6), 817–826. 10.1016/0002-8703(47)90146-4 20271989

[anec13114-bib-0040] Sclarovsky, S. , Topaz, O. , Rechavia, E. , Strasberg, B. , & Agmon, J. (1987). Ischemic ST segment depression in leads V2‐V3 as the presenting electrocardiographic feature of posterolateral wall myocardial infarction. American Heart Journal, 113(5), 1085–1090. 10.1016/0002-8703(87)90916-1 3578000

[anec13114-bib-0041] Shah, M. , Maludum, O. , Bhalla, V. , De Venecia, T. A. , Patil, S. , Curet, K. , Chinualumogu, N. , Pressman, G. S. , & Figueredo, V. M. (2016). QRS duration and left ventricular ejection fraction (LVEF) in non‐ST segment elevation myocardial infarction (NSTEMI). International Journal of Cardiology, 221, 524–528. 10.1016/j.ijcard.2016.07.028 27414734

[anec13114-bib-0042] Simes, R. J. , Topol, E. J. , Holmes, D. R., Jr. , White, H. D. , Rutsch, W. R. , Vahanian, A. , Simoons, M. L. , Morris, D. , Betriu, A. , & Califf, R. M. (1995). Link between the angiographic substudy and mortality outcomes in a large randomized trial of myocardial reperfusion. Importance of early and complete infarct artery reperfusion. GUSTO‐I investigators. Circulation, 91(7), 1923–1928. 10.1161/01.cir.91.7.1923 7895348

[anec13114-bib-0043] Sztajzel, J. , & Urban, P. (2000). Early and late Q wave regression in the setting of acute myocardial infarction. Heart, 83(6), 708–710. 10.1136/heart.83.6.708 10814637 PMC1760867

[anec13114-bib-0044] Taskesen, T. , Kaya, I. , Alyan, O. , Karadede, A. , Karahan, Z. , & Altintas, B. (2014). Prognostic value of the intermediate QRS prolongation in patients with acute myocardial infarction. La Clinica Terapeutica, 165(2), e153–e157. 10.7471/CT.2014.1700 24770825

[anec13114-bib-0045] Terkildsen, J. R. , Crampin, E. J. , & Smith, N. P. (2007). The balance between inactivation and activation of the Na+‐K+ pump underlies the triphasic accumulation of extracellular K+ during myocardial ischemia. American Journal of Physiology. Heart and Circulatory Physiology, 293(5), H3036–H3045. 10.1152/ajpheart.00771.2007 17873015

[anec13114-bib-0046] Thygesen, K. , Alpert, J. S. , Jaffe, A. S. , Chaitman, B. R. , Bax, J. J. , Morrow, D. A. , White, H. D. , & Executive Group on behalf of the Joint European Society of Cardiology (ESC)/American College of Cardiology (ACC)/American Heart Association (AHA)/World Heart Federation (WHF) Task Force for the Universal Definition of Myocardial Infarction . (2018). Fourth universal definition of myocardial infarction (2018). Journal of the American College of Cardiology, 72(18), 2231–2264. 10.1016/j.jacc.2018.08.1038 30153967

[anec13114-bib-0047] Tomcsanyi, J. , Bozsik, B. , Zsoldos, A. , & Simor, T. (2012). Isolated spontaneous septal myocardial infarction. Journal of Electrocardiology, 45(3), 280–282. 10.1016/j.jelectrocard.2011.07.013 21908000

[anec13114-bib-0048] Topal, D. G. , Lønborg, J. , Ahtarovski, K. A. , Nepper‐Christensen, L. , Fakhri, Y. , Helqvist, S. , Holmvang, L. , Høfsten, D. , Køber, L. , Kelbæk, H. , Vejlstrup, N. , & Engstrøm, T. (2020). Early Q‐wave morphology in prediction of reperfusion success in patients with ST‐segment elevation myocardial infarction treated with primary percutaneous coronary intervention ‐ a cardiac magnetic resonance imaging study. Journal of Electrocardiology, 58, 135–142. 10.1016/j.jelectrocard.2019.12.011 31869764

[anec13114-bib-0049] Topal, D. G. , Lønborg, J. , Ahtarovski, K. A. , Nepper‐Christensen, L. , Helqvist, S. , Holmvang, L. , Pedersen, F. , Clemmensen, P. , Saünamaki, K. , Jørgensen, E. , Kyhl, K. , Ghotbi, A. , Schoos, M. M. , Göransson, C. , Bertelsen, L. , Høfsten, D. , Køber, L. , Kelbæk, H. , Vejlstrup, N. , & Engstrøm, T. (2017). Association between early Q waves and reperfusion success in patients with ST‐segment‐elevation myocardial infarction treated with primary percutaneous coronary intervention: A cardiac magnetic resonance imaging study. Circulation. Cardiovascular Interventions, 10(3), e004467. 10.1161/CIRCINTERVENTIONS.116.004467 28264870

[anec13114-bib-0050] Yusuf, J. , Das, D. , Mukhopadhyay, S. , & Tyagi, S. (2018). Correlation of QRS duration with myocardial blush grade as a marker of myocardial reperfusion in primary percutaneous coronary intervention. Indian Heart Journal, 70(Suppl 3), S359–S364. 10.1016/j.ihj.2018.10.412 30595289 PMC6310739

[anec13114-bib-0051] Zafrir, B. , Zafrir, N. , Gal, T. B. , Adler, Y. , Iakobishvili, Z. , Rahman, M. A. , & Birnbaum, Y. (2004). Correlation between ST elevation and Q waves on the predischarge electrocardiogram and the extent and location of MIBI perfusion defects in anterior myocardial infarction. Annals of Noninvasive Electrocardiology, 9(2), 101–112. 10.1111/j.1542-474X.2004.92513.x 15084206 PMC6932644

[anec13114-bib-0052] Zhong, W. W. , Blue, M. , & Michaels, A. D. (2019). Acute isolated right ventricular infarction: Unusual presentation of anterior ST‐segment‐elevation myocardial infarction. Texas Heart Institute Journal, 46(2), 151–154. 10.14503/THIJ-17-6581 31236085 PMC6555289

